# Alcohol Use Disorders and Suicidal Behaviour: A Narrative Review

**DOI:** 10.62641/aep.v53i1.1772

**Published:** 2025-01-05

**Authors:** Matteo Lupi, Stefania Chiappini, Alessio Mosca, Andrea Miuli, Ilenia Di Muzio, Carlotta Marrangone, Tommaso Piro, Francesco Semeraro, Mirko Alfonsi, Livia Miotti, Maria Carlucci, Alessandro Carano, Gilberto Di Petta, Domenico De Berardis, Umberto Volpe, Giovanni Martinotti

**Affiliations:** ^1^NHS, Mental Health Department, Psychiatric Service of Diagnosis and Treatment, “Madonna del Soccorso” Hospital, AST Ascoli Piceno, 63074 San Benedetto del Tronto, Italy; ^2^UniCamillus International Medical University, 00131 Rome, Italy; ^3^Department of Neuroscience, Imaging and Clinical Sciences, University “G. d’Annunzio”, 66013 Chieti-Pescara, Italy; ^4^Unit of Clinical Psychiatry, Department of Clinical Neurosciences/DIMSC, School of Medicine, Polytechnic University of Marche, 60121 Ancona, Italy; ^5^Catholic University of the Sacred Heart, 00168 Rome, Italy; ^6^Studi Cognitivi, Cognitive Psychotherapy School and Research Center, 63074 San Benedetto del Tronto, Italy; ^7^NHS, Mental Health Department, Psychiatric Service of Diagnosis and Treatment, Santa Maria delle Grazie Hospital, ASL 2, 80134 Naples, Italy; ^8^NHS, Head of Mental Health Department, ASL Teramo, 64100 Teramo, Italy

**Keywords:** suicide, suicide attempts, suicidal behaviour, alcohol use disorder

## Abstract

**Background:**

Alcohol Use Disorders (AUD), affective disorders, and personality disorders are among the most prevalent mental health conditions observed in individuals exhibiting suicidal behavior, encompassing both completed and attempted suicides. A robust association between AUD and suicidal behavior has been established through retrospective and prospective cohort studies. Research on the relationship between alcohol consumption and self-harm has predominantly focused on Western and high-income countries, whereas approximately one-third of the global population, including half of the world's countries, lacks accessible suicide data. This study aims to present an updated review of empirical evidence regarding the risk of suicide associated with AUD in both developed and developing nations.

**Methods:**

We identified published meta-analyses, reviews, systematic reviews, randomized controlled trials, clinical studies, clinical trials, controlled clinical trials, observational studies, and case reports written in English and published between January 2004 and June 2024. Our search yielded a total of 312 papers. After reviewing titles and abstracts, 232 articles were excluded from the initial records. Following full-text review of the remaining 80 articles, a qualitative synthesis was conducted, highlighting the most representative 41 papers for inclusion in this overview.

**Results:**

Our analysis indicates that alcohol abuse is a significant risk factor for all forms of suicidal behavior. Alcohol consumption functions as both a predisposing and precipitating factor, contributing to maladaptive behaviors in both developing and developed countries. The clinical condition is exacerbated by alcohol use, which in turn increases the risk of suicide.

**Conclusions:**

Further research is essential to develop targeted psychological and pharmacological interventions aimed at preventing and treating these conditions, with the goal of reducing the risk of suicidal behavior associated with AUD. In developing countries, integrating public health and clinical strategies is crucial for effectively addressing suicide prevention.

## Introduction 

Based on data from the World Health Organization (WHO) in 2022, the harmful use of alcohol leads to approximately 3 million deaths worldwide each year, accounting for 5.3% of all deaths. Alcohol-related mortality and disability occur at a relatively early age, with around 13.5% of total deaths among individuals aged 20–39 years being attributable to alcohol [1].

Alcohol Use Disorders (AUD), affective disorders, and personality disorders are the most observed mental illnesses in individuals with suicidal behaviour, including both completed and attempted suicides [2, 3, 4, 5, 6, 7]. There is a strong connection between AUD and suicidal behavior, as supported by retrospective and prospective cohort studies [8, 9, 10] as well as post-mortem psychological autopsy studies [11].

Alcohol abuse has deep effects on mood both during periods of active intake and abstinence, which may contribute to existing risks. Additionally, increased alcohol-related impulsivity is another aspect that needs to be considered [12].

Furthermore, psychiatric disorders such as psychosis, mood disorders, anxiety disorders, and a general predisposition to stress can increase the risk of suicidal behavior [5].

The investigation into self-inflicted harm and the consumption of alcohol has primarily focused on Western and advanced nations, while approximately one-third of the global population, including half of the countries worldwide, lacks accessible suicide data. These countries primarily consist of developing nations in Asia, Africa, and South America [13, 14].

In developing countries, reports of suicides, which primarily rely on forensic data, often emphasize challenges within interpersonal relationships, emotional conflicts, domestic disputes, and financial factors. Mental disorders and alcohol usage are seldom mentioned or only implicated in a limited number of cases. This stands in stark contrast to observations made in advanced countries, where around 90% of individuals dead by suicide have been diagnosed with a psychiatric disorder [15]. Conversely, AUD represents a significant and escalating issue in developing regions, seemingly unrelated to suicidal behavior [16, 17, 18].

Despite the under-reporting and inadequate risk analysis of suicide, most suicides globally take place in developing countries such as China, India, and others. These statistics emphasize the significant prevalence of this issue and the pressing requirement for the implementation of suicide prevention strategies.

The aim of this study is to provide an updated empirical review regarding the risk of suicide linked to AUD in developed and developing countries. Additionally, it aims to critically examine the pertinent literature concerning the association between alcohol abuse and suicidal behavior in developing countries. Lastly, the study intends to give an overview of the available treatment and prevention strategies for individuals who present with both alcohol abuse and suicidal behavior.

## Methods 

### Search Strategy

We conducted a comprehensive search on MEDLINE/PubMed using the following keywords: suicide, suicide attempts, suicidal behavior, and AUD. The search included published meta-analyses, reviews, systematic reviews, randomized controlled trials, clinical studies, clinical trials, controlled clinical trials, observational studies, and case reports. The search period was between May and June 2024, aiming to identify relevant articles published from January 2004 to June 2024. This search yielded a total of 312 results. To ensure thoroughness, we expanded our search by consulting additional databases, including Scopus, Google Scholar, and PsychInfo, to identify any studies not captured in the initial search.

### Study Selection

**Fig. 1.**
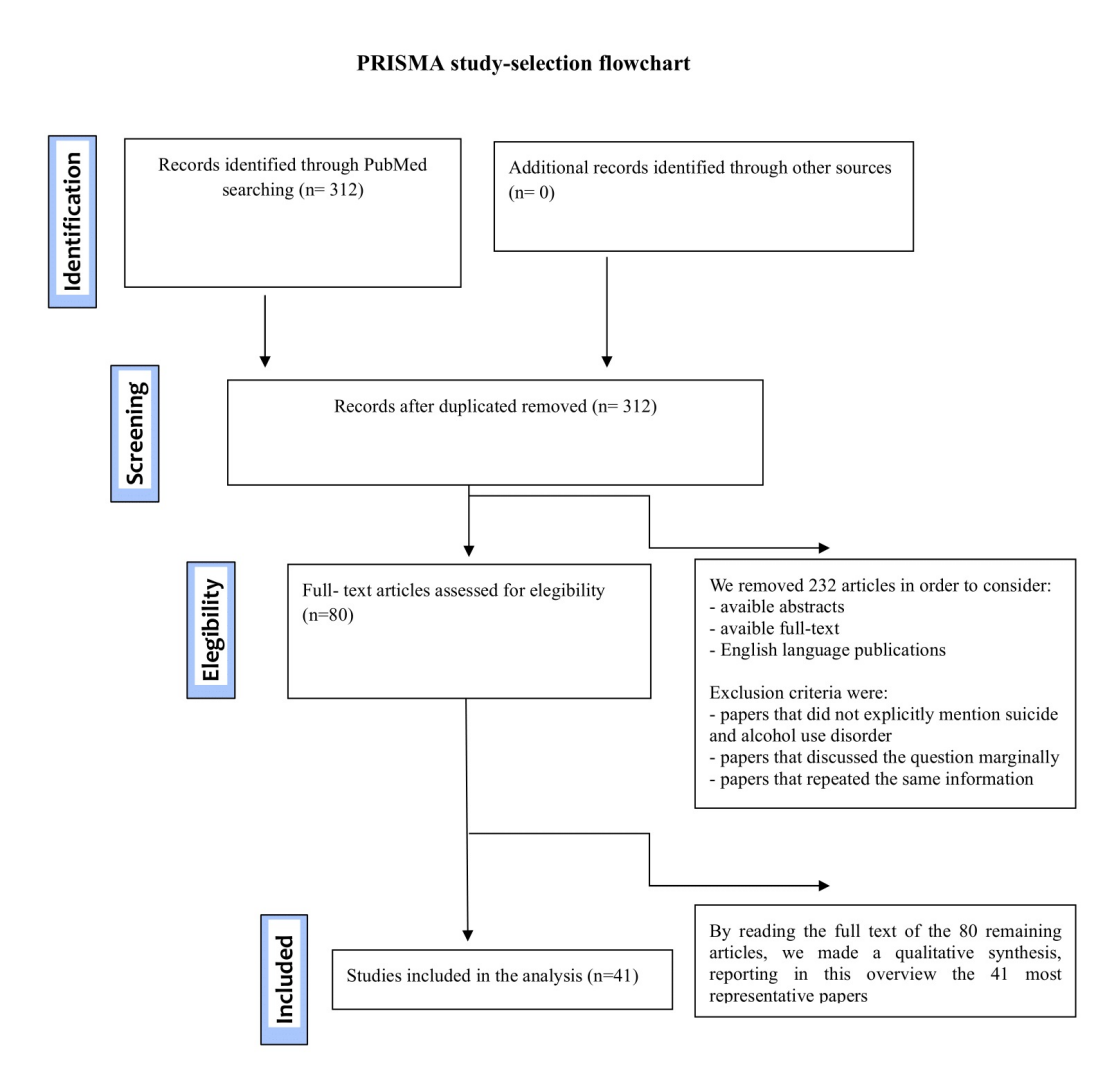
**PRISMA study-selection flowchart**.

The titles and abstracts of all articles were rigorously evaluated. The inclusion of papers in the final review was determined through discussions between pairs of authors. In cases of disagreement, resolution was achieved during a steering group meeting involving all authors. Inclusion criteria required that papers had accessible abstracts, available full texts, and were published in the English language. We excluded 232 articles after reviewing titles and abstracts, removing papers that did not explicitly mention suicide and AUD, discussing the topic only marginally, or presenting redundant information. After reviewing the full texts of the remaining 80 articles, we conducted a qualitative synthesis, ultimately selecting the 41 most representative papers for inclusion in this overview. The selected studies were categorized according to the different types of suicidal behavior associated with alcohol use, specifically AUD and completed suicide, as well as AUD and attempted suicide. Additionally, a distinction was made between studies conducted in developed countries and those from developing nations (Fig. 1).

## Results

The selected studies published from January 2004 to June 2024 are described and classified in Table 1 (Ref. [7, 9, 10, 19, 20, 21, 22, 23, 24, 25, 26, 27, 28, 29, 30, 31, 32, 33, 34, 35, 36, 37, 38, 39, 40, 41, 42, 43, 44, 45, 46, 47, 48, 49, 50, 51, 52, 53, 54, 55]).

**Table 1.** **Studies selected (January 2004–June 2024)**.

	Type of study	Country	Population	Sample size	Completed suicide	Attempted suicide	Major findings
Borges *et al*. [27], 2004	Case-control study	USA, Canada, Mexico, Australia	Selected	102	no	yes	Alcohol Use Disorders (AUD) and suicide attempt positively related
Cherpitel *et al*. [9], 2004	Review	Developed countries	General	Not specified	yes	yes	Acute alcohol use is linked to suicidal behavior
Conner *et al*. [19], 2004	Empirical review	Developed countries	General	Not specified	yes	no	Aggression/impulsivity, severe alcoholism, negative affect, and hopelessness are predisposing factors to suicide among alcoholics
Pirkola *et al*. [55], 2004	Review	Developed countries	General	Not specified	yes	yes	Alcohol dependence is related to a major risk of suicidal behavior
Wilcox *et al*. [10], 2004	Review of cohort studies	Developed countries	Selected	1690	yes	no	AUD is associated with completed suicide, principally in women
Bilban *et al*. [22], 2005	Retrospective study	Slovenia	Selected	508	yes	no	Alcohol was present in 68.8% of all suicides committed
Hutchinson [53], 2005	Retrospective study	Trinidad and Tobago	Selected	Not specified	yes	no	Alcohol consumption was significantly correlated to suicide
Van der Hoek and Konradsen [43], 2005	Case-series	Sri Lanka	Selected	239	yes	no	Alcohol dependence major risk factor for completed suicide
Akechi *et al*. [21], 2006	Cohort study	Japan	General	>10,000	yes	no	Proportionality between alcohol consumption and risk of suicide
Brady [25], 2006	Review	NA	General	Not specified	yes	yes	AUD predisposes to suicidal behavior
Measey *et al*. [23], 2006	Retrospective study	Australia (Northern)	Selected	Not specified	yes	no	72% of suicide reports presented alcohol abuse conducts
Sher [26], 2006	Review	Developed countries	General	Not specified	yes	yes	Alcoholism is related with a significant risk of suicidal behavior
Tran Thi Thanh *et al*. [48], 2006	Case-series	Vietnam	Selected	2280	no	yes	AUD associated with suicidal thoughts
Hawton *et al*. [7], 2009	Case-control study	UK	Selected	7344	no	yes	Alcohol involved in 54.9% of appraised episodes of self-harm
Galaif *et al*. [20], 2007	Review	Developed countries	NA	NA	yes	yes	Alcohol use and depression are the most significant risk factors for both attempted and completed suicide among teenagers
Hong *et al*. [47], 2007	Case-series	China	Selected	454	no	yes	Suicidal ideation was more frequent among female sex workers with AUD
Li [42], 2007	Case-series	Taiwan	Selected	11,837	yes	yes	Approximately 50% of deliberate self-harm incidents were linked to alcohol use
Makhija [29], 2007	Review	USA	Selected	Not specified	yes	yes	The adolescents who abuse alcohol or substances show a higher rate of suicidality than those who do not; abuse appears to be a risk factor for eventual suicidality
Nojomi *et al*. [51], 2007	Case-control study	Iran	General	2300	no	yes	Long-term alcohol use is an independent predictor of suicide attempt
Palacio *et al*. [24], 2007	Case-control study	Colombia	Selected	108	yes	no	AUD found in 43% of suicides
Razvodovsky [45], 2009	Retrospective study	Belarus	General	64,162	yes	no	A positive association between alcohol consumption and suicide has been observed, influenced by binge drinking
Park *et al*. [49], 2008	Case-control study	Korea	General	11,523	yes	no	AUD is a significant risk factor for completed suicide
Pérez-Olmos *et al*. [44], 2008	Case-series	Colombia	Selected	156	no	yes	Suicide attempts were related with a history of alcohol use
Aseltine RH, Jr *et al*. [36], 2009	Longitudinal study	USA	Selected	32,217	no	yes	Heavy episodic alcohol consumption was strongly associated with self-reported suicide attempts
Pridemore and Snowden [54], 2009	Retrospective study	Slovenia	Selected	Not specified	yes	no	The authors show that a new national policy restricting alcohol availability has contributed to a reduction in male suicides
Schilling *et al*. [28], 2009	Cohort study	USA	Selected	3954	no	yes	Binge drinking is significantly correlated with self-reported suicide attempts
Boenisch *et al*. [35], 2010	Case-series	Germany	General	1921	no	yes	Individuals with AUD constitute a high-risk group for multiple suicide attempts. The need of suicide prevention is recommended
Borges G and Loera CR [52], 2010	Review	Developed and developing countries	General	Not specified	yes	yes	The authors highlight the causal role of alcohol and drug use disorders in suicidal behavior
Oquendo *et al*. [32], 2010	Retrospective study	USA	Selected	1643	no	yes	Bipolar individuals with comorbid AUD were at increased risk for suicide attempt compared to individuals without AUD
Bhattacharjee *et al*. [50], 2012	Cohort study	India	Selected	200	no	yes	Alcoholism raises the suicide rate
Klimkiewicz *et al*. [34], 2012	Case-series	Poland	Selected	113	no	yes	Around 66% of the patients reported their gravest suicide attempt as concurrent with a period of heavy drinking
Morin *et al*. [30], 2013	Case-control study	Sweden	Selected	103	no	yes	A significant correlation between AUD and hospitalization due to suicide attempts was observed in both males and females
Swahn *et al*. [33], 2013	Cohort study	USA	General	16,410	no	yes	Both early onset alcohol use and heavy drinking are statistically correlated with suicide attempts among young people
Mcmanama O’Brien *et al*. [31], 2014	Longitudinal study	USA	Selected	143	no	yes	A significant interaction effect was found such that in adolescents with suicidal ideation and low levels of depression, a higher frequency of alcohol consumption was associated with an increased likelihood of a suicide attempt
Richards *et al*. [37], 2020	Retrospective study	USA	Selected	43,706	no	yes	Among patients who reported suicidal ideation, the risk of acting out was higher at all levels of alcohol consumption, particularly at high levels
Orpana *et al*. [46], 2021	Systematic review	Canada, USA	General	Not specified	yes	no	Higher levels of alcohol sales or consumption were associated with higher suicide rates. Attributable fraction studies esteem that ¼ of suicide deaths in Canada are attributable to alcohol misuse
Ahuja *et al*. [39], 2021	Retrospective study	USA	General	13,867	no	yes	High odds of suicidal attempts for adults are correlated with early age of alcohol initiation, with the highest odds for those who began drinking at or before the age of 14
Kim *et al*. [40], 2021	Survey	Korea	Selected	5982	no	yes	Alcohol use and impulsivity might lead from suicidal ideation to suicidal attempt
Edwards *et al*. [38], 2022	Longitudinal study	Sweden	General	2,229,619	no	yes	AUD appears to be a significant predictor of suicide attempts, even when accounting for the presence of the other psychiatric disorders
Ledden *et al*. [41], 2022	Survey	England	Selected	14,949	no	yes	The authors emphasized a linear relationship between AUD and suicide attempt, suicidal ideation, and self-harm

## Developed Countries

### Alcohol Use Disorders (AUD) and Completed Suicide 

According to Hasin [56] (2020), the lifetime mortality rate for suicide among individuals with alcohol dependence is estimated to be 18%. Earlier research by Roy and Linnoila [57] (1986) found that 2.2% of alcohol-dependent patients treated in outpatient settings and 3.4% of those treated in inpatient settings die by suicide. Recent studies, such as one by Orpana *et al*. [46], have identified ecological correlations in Canada, showing that higher alcohol sales or consumption levels are associated with higher suicide rates, with 25% of suicide deaths attributable to alcohol misuse [45]. Similarly, a study by Wilcox *et al*. [10] (2004) indicated a stronger association between AUD and completed suicide, particularly among women.

Gender differences in suicide risk among individuals with AUD have been corroborated by findings from the National Comorbidity Survey (NCS) in the United States and a case-control study conducted by Conner *et al*. [58] in New Zealand in 2003. These studies revealed that, even after controlling for depression, women with alcoholism had a higher risk of suicide compared to the general population [58, 59, 60]. Individuals who die by suicide often exhibit patterns of heavy alcohol consumption, more alcohol-related medical issues, earlier initiation of alcohol use, and longer durations of alcoholism [19, 61, 62, 63, 64]. Stack’s [65] (2000) literature review found that out of 84 studies conducted across 17 countries, 55 reported a positive correlation between alcohol consumption and suicide rates.

Young individuals appear to be particularly vulnerable to completed suicide related to alcohol use. For instance, Galaif *et al*. [20] found that alcohol use and depression were the primary risk factors for both attempted and completed suicide in teenagers, with binge drinking being a critical factor [66, 67, 68, 69]. Furthermore, a large cohort study conducted in Japan indicated that middle-aged Japanese men who were heavy drinkers had an elevated risk of subsequent suicide [21].

Psychological autopsy studies have revealed that over 80% of individuals who died by suicide had experienced affective (mood) and addictive disorders. Retrospective analysis found that alcohol abuse or dependence was present in 15–70% of cases among unselected populations of suicide victims [22, 23, 70, 71]. Additional studies have highlighted a higher frequency of interpersonal losses among individuals with alcohol dependence compared to those who were depressed before their death [24, 72]. Earlier research by Kendall [73] (1983) and Lester [74] (1993) proposed that alcohol abuse diminishes self-esteem, thereby increasing the risk of suicide.

Comorbidity has emerged as a significant risk factor for suicide among individuals with alcohol dependence. In particular, alcohol dependence comorbid with major depression carries a substantially higher risk of suicide than either disorder alone [75]. Cornelius *et al*. [76] (1995) examined patients in an urban psychiatric facility and found that depressed alcoholics exhibited a greater tendency toward suicide compared to individuals with either depression or alcohol dependence alone. This suggests a synergistic effect of the two diagnoses, with the depressive effects of alcohol compounding the risk of suicidal behavior [76, 77]. These findings are supported by various studies reporting that 45–70% of individuals who die by suicide and have alcoholism also experience major depression toward the end of their lives [60, 61, 75].

Impulsive and aggressive traits are frequently identified as risk factors for suicide among individuals with alcoholism [78, 79]. Neurobiological studies have suggested that differences in serotonergic and noradrenergic neurotransmission, which are involved in AUD, may contribute to the biology of impulsivity, thus increasing the likelihood of suicide [80, 81]. This implies a potential shared genetic predisposition between alcohol misuse and completed suicide [25].

Regarding the act of suicide itself, alcohol as an intoxicating substance impairs judgment, heightens impulsivity, and facilitates suicidal behavior [82, 83]. Alcohol has a biphasic effect on emotions: lower doses may temporarily alleviate negative effects, whereas higher doses induce depressive effects on the central nervous system [84]. Mechanisms by which alcohol elevates the risk of suicide include increased psychological distress, activation of suicidal thoughts, and cognitive narrowing that limits the ability to generate and implement alternative coping strategies [26]. Additionally, intoxicated individuals are more likely to use more lethal means of suicide, such as firearms [85]. However, the precise role of intoxication in the final act of suicide remains incompletely understood.

### Alcohol Use Disorders (AUD) and Suicide Attempts 

Alcohol abuse or dependence has been found in 27–72% of individuals who have attempted suicide [27, 86, 87, 88, 89]. Among patients with alcohol dependence, 16–29% have attempted suicide at some point in their lives [90, 91, 92]. A study of 31,953 students in the United States revealed significant correlations between binge drinking, heavy episodic drinking, and self-reported suicide attempts [28]. Additionally, among alcohol-dependent individuals who have attempted suicide, approximately 45% exhibited subsequent episodes of self-harm during a follow-up period of 12–20 months [88]. Half of the alcohol-dependent individuals displaying suicidal behavior were also diagnosed with a mental illness [88, 91].

A study by Haw *et al*. [89] (2001) found that men who misuse alcohol, live alone, are unemployed, and have medical problems are more likely to have attempted suicide. Other associated risk factors include lower socioeconomic status, younger age, earlier onset of alcohol problems, and a history of childhood abuse [29, 93]. Alcohol abuse also elevates the risk of suicidal behavior in older adults. Morin *et al*. [30] (2013) found that the lifetime prevalence of AUD was significantly higher (26%) among hospitalized older adults who had attempted suicide compared to a comparison group (4%) (Odds Ratio (OR): 10.5).

In adolescents, alcohol use can accelerate the transition from suicidal thoughts to actual suicide attempts, particularly in those with low-severity depression [31]. Oquendo *et al*. [32] (2010) found that over half (54%) of participants with bipolar disorder (BD) also had AUD, with those having both conditions at a higher risk for suicide attempts (OR: 2.25). Similarly, Rojas *et al*. [94] (2014) demonstrated a significant association between post-traumatic stress disorder (PTSD) and alcohol dependence, which was linked to a higher likelihood of suicidal thoughts and attempts. Aggressive and impulsive traits are additional risk factors for suicidal behavior in individuals with alcoholism and other substance use disorders (SUD) [95]. A study of alcohol-dependent individuals undergoing detoxification found that those with a history of suicide attempts exhibited a higher incidence of aggressive behavior and scored higher on aggression/impulsivity measures [84]. Acute alcohol intoxication can exacerbate suicidal tendencies in vulnerable individuals and increase the lethality of attempts [33, 34, 35].

A recent study involving 16,410 high school students found a strong association between early alcohol use (before age 13), binge drinking, physical fighting, and suicide attempts [33]. Screening data from 32,217 students aged 11–19 years across 225 USA schools similarly revealed a significant association between heavy episodic drinking and self-reported suicide attempts [36]. Another study of 113 patients reported that over two-thirds of major suicide attempts occurred during periods of high alcohol use [34]. An analysis of 1921 suicide attempts in a large German city found that 17% of attempts involved individuals diagnosed with AUD, and 32% occurred under acute alcohol consumption [35].

The link between alcohol dependence and suicidal ideation has been confirmed by studies such as Grant and Hasin’s [96] (1999), which specifically associated suicidal thoughts and communication with alcohol dependence. Richards *et al*. [37] (2020) showed that patients reporting suicidal ideation had a significantly higher risk of suicide attempts, particularly when combined with heavy episodic drinking. Women with AUD are at greater risk during adolescence, while the risk for men peaks around age 30. Earlier onset of AUD is also associated with higher odds of attempted suicide. Impulsivity, often exacerbated by alcohol use, contributes to the transition from suicidal thoughts to attempts [38, 39, 40]. In a representative sample of the general population in England, studies suggested that when alcohol use severely disrupts daily functioning, it strengthens the relationship between alcohol consumption and suicide-related outcomes, emphasizing the need to address addiction behaviors in addition to drinking patterns [41].

The emerging problem of extreme weight control behaviors, such as binge drinking to compensate for binge eating, has also been noted among young adults. A survey in Italy involving 4275 healthy subjects found a significant correlation between drunkenness and binge drinking [97]. Additionally, Martinotti *et al*. [98] found that drinking habits were strongly associated with the use of new psychoactive substances (NPS), particularly in urban areas. In a study on alcohol consumption and NPS use in Italy, a questionnaire administered to 206 psychiatric patients aged 18–26 and 2615 healthy subjects showed that alcohol consumption was more frequent in the healthy population compared to those with mental illness [99].

The COVID-19 pandemic had a profound impact on individuals with SUD and AUD. A study conducted during the pandemic involving 153 drug-addicted patients demonstrated a positive correlation between craving and symptoms of depression, pessimistic ideation, anxiety, and traumatic stress [100].

## Developing Countries

### Alcohol Use Disorders (AUD) and Completed Suicide

The data from the WHO mortality database highlights that a significant proportion of global suicides, approximately 85%, occur in low- and middle-income countries [101]. However, religious, legal, and cultural factors often lead to underreporting of these deaths in many regions [102]. Suicide registration practices vary greatly, with some Islamic countries considering suicide a criminal act, which affects accurate data collection [103]. For example, a population-based study in India revealed that enhanced data collection methods uncovered suicide rates that were nine times higher than officially reported figures [104]. Similarly, alcohol use is often underestimated and underreported, particularly in countries with limited access to mental health professionals due to similar religious, legal, and cultural constraints [105].

Globally, studies frequently do not report AUD and other psychiatric disorders as precipitating or predisposing factors for suicide [17, 18]. Instead, suicide is often attributed to external causes such as domestic or interpersonal issues, or work-related problems. Nevertheless, in countries where data on AUD and suicidal behaviors are available, a consistent association is observed. For example, in the Pondicherry region of India, alcohol consumption has been strongly correlated with high suicide rates, contributing to one of the highest levels of both alcohol use and completed suicides in the country [106]. A population-based control study in India also found that approximately 34% of individuals who died by suicide were diagnosed as alcoholics [107, 108].

Similar findings are observed across other regions, such as Taiwan, where substance and alcohol use were present in 40–50% of suicide cases, and Sri Lanka, where alcohol use was a significant factor in suicide cases [42, 43, 109]. In South America, recent studies indicate that alcohol consumption was present in 49% of suicide attempts and 43% of completed suicides in Colombia [24, 44]. In Belarus, binge drinking has been identified as a contributing factor to the positive association between alcohol use and suicide [45]. Furthermore, the relationship between AUD and suicide is also evident among low-income ethnic and racial groups in developed countries, such as American Indians, Alaskan Natives, and residents of Australia’s Northern Territory, where alcohol consumption and suicide rates are higher compared to the general population [110, 111].

### Alcohol Use Disorders (AUD) and Suicide Attempts

Research consistently demonstrates the link between alcohol consumption and suicide attempts. For example, a recent study involving 200 Indian patients who survived a suicide attempt found that 17% had consumed alcohol before their attempt [46]. In China, a study among female sex workers showed a strong association between alcohol intoxication and suicidal ideation or attempts [47]. Similar patterns are observed across various countries; in Vietnam, suicidal thoughts were associated with alcohol and sedative use [48]; in South Korea, early-onset alcoholism was specifically linked to higher rates of suicide attempts compared to late-onset alcoholism [49, 50, 112].

Even in regions where alcohol consumption is typically low due to religious restrictions, such as the Middle East, studies show that factors like younger age, female gender, a history of mental disorders, and lifetime alcohol use are independent predictors of suicide attempts. In Tehran, Iran, these factors were significantly associated with suicide attempts in a large sample [51].

In Africa, a study in Ethiopia found that individuals with problematic drinking behaviors had a higher number of lifetime suicide attempts compared to those without drinking problems [113]. Similarly, in South Africa, 24% of subjects admitted to an alcohol rehabilitation center had a history of suicide attempts, with risk factors such as female gender, being white, being unmarried, younger age, and early onset alcohol misuse [114]. In contrast, a study in India found that female suicide attempters exhibited minimal alcohol consumption [115].

In Latin America, AUD has been linked to a heightened risk of suicide ideation and attempts. For example, in Mexico, individuals with AUD had odds ratio ranging from 2.0 to 2.5 for ideation and 2.6 to 3.7 for attempts. Moreover, those who consumed alcohol prior to a suicide attempt had significantly increased odds, with ratio ranging from 6.2 to 9.6 [52]. Similar findings have been reported in Brazil [116], Argentina [117], Trinidad and Tobago [53], and in Mexico’s Hidalgo region, where women who were heavy drinkers had a 1.57 times higher risk of suicidal ideation compared to non-drinkers [118].

This data highlights the complex and global nature of the relationship between alcohol use and suicidal behaviors, with varying risk factors and patterns emerging across different cultural and socioeconomic contexts.

## Prevention Strategies 

Numerous countries have instituted national suicide prevention programs designed to target high-risk populations and reduce overall suicide rates [7]. A Cochrane review indicated that interventions aimed at mitigating problem drinking might lower suicide rates and the incidence of suicide attempts, though quantifying the precise impact of these interventions remains difficult [119]. In Slovenia, the enactment of a national policy limiting alcohol availability was associated with a substantial decrease in monthly suicide rates [54].

Martinotti *et al*. [100] (2020) investigated the effects of stringent quarantine measures during the COVID-19 pandemic on individuals with SUD. Their study revealed that restricted substance availability impacted the development of cravings, and intensive residential treatment, combined with severe restrictions on substance acquisition, had notable therapeutic effects [100].

There is limited research on pharmacological treatments for suicidal patients with alcoholism, partly because suicidality is frequently an exclusion criterion in clinical trials [120]. Only two systematic reviews have specifically addressed the psychopharmacological treatment of suicidal patients with alcoholism [121, 122]. These studies suggest that selective serotonin reuptake inhibitors (SSRIs) can significantly alleviate depressive symptoms, including suicidal ideation, and reduce alcohol consumption in individuals with comorbid depression and AUD.

A pilot study proposed a cognitive-behavioral treatment protocol for adolescents with co-occurring AUD and suicidality, demonstrating promising results in decreasing alcohol use and suicidal ideation among participants [123].

Pirkola *et al*. [55] (2004) outlined management principles for patients at risk of suicide who are also alcohol-dependent: (1) Immediate evaluation for suicide risk is essential when patients express suicidal thoughts while intoxicated, particularly if additional risk factors or recent adverse life events are present. Continuous monitoring during intoxication may be necessary. (2) The presence of symptoms of co-occurring mental disorders should alert healthcare professionals to potential suicide risk in an intoxicated patient, regardless of direct suicidal communication [55].

Cornelius *et al*. [120] (2004) recommend hospitalization and close observation for patients with alcoholism who disclose a suicide plan or intent. Sher [124] (2007) also suggests considering hospitalization for individuals with alcoholism who exhibit severe agitation, pronounced impulsivity, express hopelessness, or have a history of suicide attempts. Family education on creating a safer home environment should be conducted before discharge [124].

Suicide prevention strategies in developing countries are generally less advanced compared to those in developed countries. Sri Lanka is an exception, having implemented a specific national suicide prevention plan which led to a notable reduction in the overall suicide rate [125]. The lack of coordinated national plans and insufficient mental health services in most developing countries have hampered effective suicide prevention efforts. Consequently, Non-Governmental Organizations (NGOs), play a vital role in addressing this gap. These NGOs often function as crisis centers or hotline services, primarily staffed by volunteers, and in many cases are the sole suicide prevention agencies within their countries. The significance of these services is highlighted by the findings of a study by Marecek and Ratnayeke [126] (2001).

## Discussion 

The association between alcohol misuse and various forms of suicidal behavior is well-established. Alcohol serves as both a contributing factor due to its depressive effects and a triggering factor owing to increased impulsivity while intoxicated. However, there is a notable lack of research specifically targeting individuals with alcoholism who exhibit suicidal ideation and behaviors. Additionally, studies on AUD reveal significant variability, with limitations stemming from differences in sample characteristics, research methodologies, AUD severity, and diagnostic criteria, affecting the generalizability of the findings.

Despite substantial advancements in psychiatric treatment, global suicide rates have continued to rise over the past two decades [127]. This increase is partly due to the notable rise in suicides in many developing countries. The impact of alcohol and drug abuse on suicide rates is evident from the reviewed studies. While alcohol consumption among adults has generally decreased in most developed countries since 1980, it has risen in developing countries, former Soviet Union states, and the UK and Ireland [128]. There has been a shift towards higher rates of binge drinking among youth [129]. Wilcox and colleagues [10] (2004) observed that the association between AUD and suicide is more pronounced in women than men, a finding applicable to developing countries as well [50, 110, 115]. However, conclusions regarding developing countries are limited due to sparse data. Ramstedt [69] (2001) suggested that women with alcohol abuse issues might face a higher risk of suicidal behavior due to increased social stigmatization and diminished social integration compared to men. Gender considerations are crucial in psychiatry and should extend to substance and alcohol misuse [130]. Another neurobiological hypothesis posits that women might be more sensitive to the depressogenic effects of alcohol. To accurately assess risk levels among women, identify relevant factors, and focus prevention and intervention efforts, more data on suicide and AUD are needed. Young age and early-onset alcoholism are significant risk factors for alcohol-related completed suicides, especially among binge drinkers, in both developed and developing countries [66, 67]. Sher [124] (2007) proposed that dysregulation of the hypothalamic-pituitary-adrenal axis might contribute to both alcohol abuse and suicidal behavior in adolescents, emphasizing the need for targeted prevention strategies for this age group [55].

The role of social stressors, such as generational conflicts, romantic failures, poor physical health, and academic problems, is more pronounced in developing countries, whereas psychiatric issues are more strongly associated with suicidal behavior among alcoholics in developed countries. This discrepancy may result from an underestimation of the psychiatric burden in developing countries. In these contexts, stress factors are often perceived as environmental and sociocultural, while in individualistic Western societies, stress is frequently viewed as an internal issue. Perceived stress in specific populations is often attributed to environmental factors, contributing to mental health problems [131].

Alcohol consumption exacerbates maladaptive behaviors in both developing and developed countries, increasing the likelihood of suicide. This effect is likely mediated by increased impulsiveness and aggression, which are consistently observed in various studies [19, 79]. Moreover, individuals with AUD, particularly women, often exhibit lower levels of empathy and difficulties in recognizing and expressing their emotional states, a condition known as alexithymia. This may drive susceptible individuals to misuse alcohol as a coping mechanism for their emotional vulnerabilities. Therefore, addressing these psychopathological traits is crucial as a preventive strategy for individuals with pre-existing alcohol misuse tendencies [132].

The use of mood stabilizers and other pharmacological treatments in managing alcohol use disorder may be beneficial, particularly in reducing suicide risk [133].

## Conclusions 

Further research is necessary to develop psychological and pharmacological interventions tailored specifically for individuals with AUD who exhibit suicidal behavior. Clinicians should be aware that suicidal behavior is prevalent among individuals with alcoholism, particularly those with co-occurring AUD and affective disorders. Routine screening for suicide risk should be conducted in all patients with alcoholism, and hospitalization should be considered if there is evidence of a suicidal plan or intent.

In developing countries, an integrated approach combining public health and clinical strategies is essential for effective suicide prevention. Regions such as Southeast Asia and Africa, which encompass a substantial portion of the global population, face a shortage of mental health professionals. Collaboration with non-governmental organizations, traditional healers, and alternative medicine practitioners could enhance suicide prevention efforts.

A centralized infrastructure for national suicide prevention plans may not be feasible; instead, a more practical approach involves identifying key stakeholders and engaging them in the development and implementation of prevention strategies, while ensuring efficient resource allocation. Additionally, establishing regional centers for suicide monitoring and surveillance could facilitate the exchange of information and research findings among countries with similar cultural, social, and economic characteristics, such as those in Southeast Asia, Africa, and South America.

Developing countries should prioritize the implementation of national suicide prevention plans that are comprehensive and contextually relevant. It is also crucial to initiate targeted programs for particularly vulnerable populations, such as women and youth, and to implement strategies for identifying individuals with AUD at risk of suicide, both in developed and developing countries.

## Availability of Data and Materials

All data generated or analyzed during this study are included in this published article.
